# Capitalising on Tax Variation to Estimate the Effect of Alcohol Consumption on Edentulism

**DOI:** 10.1111/jcpe.14176

**Published:** 2025-05-06

**Authors:** Sebastian‐Edgar Baumeister, Birte Holtfreter, Thomas Kocher, Gustavo G. Nascimento

**Affiliations:** ^1^ Institute of Health Services Research in Dentistry University of Münster Münster Germany; ^2^ Department of Restorative Dentistry, Periodontology and Endodontology University Medicine Greifswald Greifswald Germany; ^3^ National Dental Research Institute Singapore, National Dental Centre Singapore Singapore; ^4^ Oral Health Academic Programme, Duke‐NUS Medical School Singapore

**Keywords:** alcohol consumption, alcohol tax, edentulism, instrumental variable

## Abstract

**Aim:**

To estimate alcohol consumption's effect on edentulism using state alcohol taxes as an instrumental variable (IV).

**Material and Methods:**

Analysis of 514,357 U.S. Behavioural Risk Factor Surveillance System participants (2003–2006, 2008, 2010, 2012) linked to state alcohol taxes. We used IV regression modelling to assess the relationship between alcohol consumption and edentulism, plus potential mediators (body mass index, dental visits) and a positive control (coronary heart disease). Robustness to imperfect exogeneity was evaluated through sensitivity analyses and falsification testing using IV analysis on individuals under the age of 16 years.

**Results:**

A 1.1‐drink increment per day was associated with a 12% higher risk of edentulism (95% confidence interval: 9%–16%). Alcohol consumption was positively associated with body mass index, dental visits and coronary heart disease. No significant effect on edentulism was observed in the negative control population (individuals aged < 16 years).

**Conclusion:**

The findings of this quasi‐experimental study suggest that alcohol consumption increases the risk of edentulism.

## 1 Introduction

Alcohol consumption is a leading global health risk, contributing to 1.8 million deaths worldwide in 2020 and disproportionately affecting young males (GBD 2020 Alcohol Collaborators 2022). Its prevalence has increased in many low‐ and middle‐income countries since the 1990s. Extensive evidence links alcohol consumption to various chronic diseases, including cancers, cardiovascular disease (CVD), liver cirrhosis and infectious diseases (Nyberg et al. 2022; WHO 2018; Wood et al. 2018).

Edentulism, the complete loss of all natural teeth, affects millions worldwide, with a global prevalence of 7% among adults aged 20 and older (Nascimento et al. 2024; WHO 2024). This burden is particularly high among older adults (> 60 years) with a 23% prevalence. Between 1990 and 2019, global edentulism cases increased by 81%, reaching over 350 million. This significant rise, driven by a global increase in prevalence, underscores a major public health challenge, particularly in low‐ and middle‐income countries. Edentulism has profound social and health consequences. It can significantly impact nutrition, speech, social interaction and self‐esteem, leading to social isolation and limited employment opportunities (Aida 2021; Elani et al. 2017; Hunter et al. 2024; Matsuyama et al. 2019). Moreover, tooth loss is associated with increased risks of CVD, cancer and premature mortality (Aminoshariae et al. 2024; Bond et al. 2022; Higham and Scannapieco 2024; Hunter et al. 2024; Matsuyama et al. 2023).

Tooth loss typically results from a lifelong history of oral diseases, mainly advanced dental caries and severe periodontal disease, but can also occur as a result of trauma, lack of dental services in resource‐poor settings or overtreatment (Aida 2021; Burt 1978; WHO 2024). The proximal causes of tooth loss, including caries, periodontitis, dental trauma and dental treatment philosophy, are mediated by modifiable factors, including oral‐health‐related behaviours (Aida 2021). For example, smoking is a known cause of periodontitis and tooth loss (Baumeister et al. 2021, 2024). Alcohol is another potentially modifiable risk factor for tooth loss. Several prospective studies have suggested that alcohol increases the risk of periodontitis (Oliveira et al. 2022; Pulikkotil et al. 2020; Wang et al. 2016). Observational studies have also shown associations with tooth loss but have produced inconsistent results (Copeland et al. 2004; Weintraub et al. 2019; Wu et al. 2011), with some studies showing an increase in tooth loss risk associated with alcohol consumption while other studies suggesting a protective association.

We employed a quasi‐experimental approach using alcohol taxes as an instrumental variable (IV) to estimate the effect of alcohol consumption on edentulism. We used repeated cross‐sectional data from the U.S. Behavioural Risk Factor Surveillance System (BRFSS).

## 2 Material and Methods

### 2.1 Data, Design and Variables

The BRFSS, a state‐based system of telephone health surveys, was established in 1984 by the U.S. Centers for Disease Control and Prevention (CDC) (Mokdad et al. 2003). It collects information on health risk behaviours, preventive health practices and selected health conditions, primarily related to chronic diseases and injuries, through telephone interviews. The survey employs a random‐digit‐dialing strategy, targeting non‐institutionalised individuals, to ensure a representative sample from all 50 states, the District of Columbia and three US territories, and includes both landline and cellular telephone interviews. We analysed pooled data from 2003 to 2006, 2008, 2010 and 2012, including individuals aged ≥ 30 years who reported alcohol consumption during the past 30 days, and linked these data to state‐level alcohol taxes based on the participants' state of residence and survey year. We restricted our sample to current (last month) alcohol drinkers to limit potential biases associated with including former drinkers who may abstain from alcohol because of ill health (Wood et al. 2018). This restriction was also based on the hypothesis that our alcohol tax IVs primarily affect alcohol consumption among current drinkers, rather than inducing initiation or abstention in our adult sample (Babor 2023).

**FIGURE 1:**
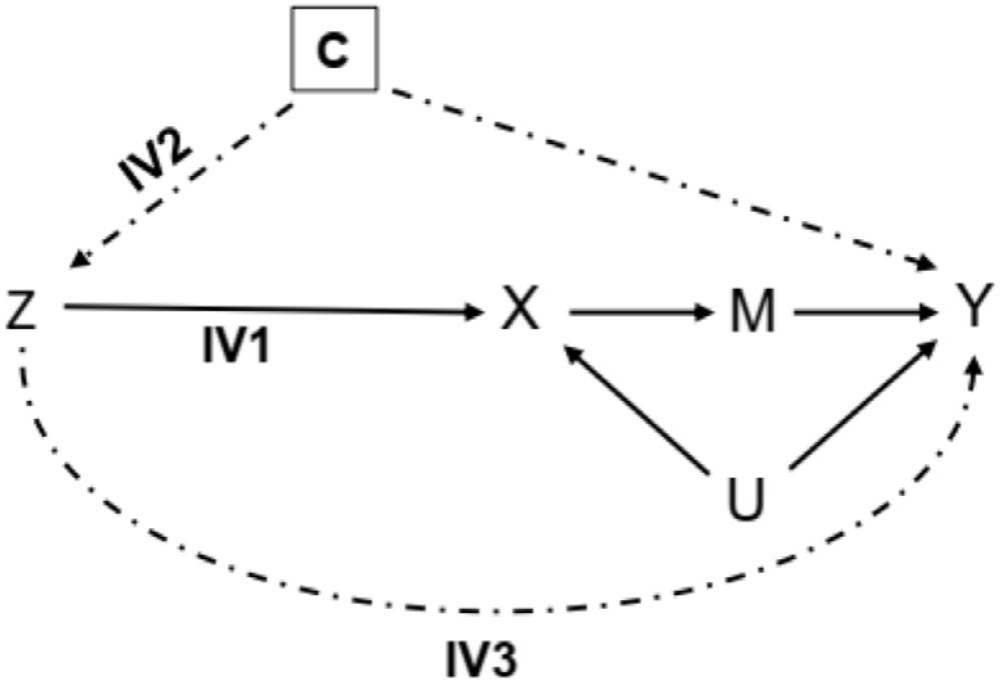
Causal graph illustrating the instrumental variable design and assumptions to estimate the effect of alcohol consumption on edentulism. Z represents the alcohol tax instrumental variable (IV). X represents the alcohol exposure. Y represents the edentulism outcome. U represents unobserved exposure–outcome confounders. M represents potential mediators, including body weight, diabetes, periodontitis, dental trauma, oral hygiene or dental attendance. IV1, IV2 and IV3 represent the relevance, conditional exchangeability and exclusion restriction assumptions. Dashed arrows represent where there should be an absence of effect for IV2 and IV3 assumptions to be valid.

We used edentulism (complete tooth loss) as the outcome variable (Table 1). The exposure variable was ‘drinks per day’, defined as the average number of standard alcoholic drinks consumed per day over the past month (CDC Alcohol Program 2024). We used taxes for beer, wine and spirits as instruments for exposure variable (Figure 1). These taxes represent year‐ and state‐specific excise taxes per gallon (5% beer, 12% wine, and 40% spirits by alcohol volume), adjusted for inflation to the 2012 Consumer Price Index, obtained from the Alcohol Policy Information System (National Institute on Alcohol Abuse and Alcoholism 2024). Research has demonstrated that increased alcohol taxes reduce alcohol consumption in the U.S. population (Blanchette et al. 2020), and many studies have used state‐level alcohol taxes as IVs for individual‐level alcohol consumption (Bray 2005; Cook and Moore 1993; French and Maclean 2006; Terza 2002). Our IV models were adjusted for potential IV‐outcome confounders (C in Figure 1), including age, sex, race/ethnicity, educational attainment and smoking status. Race/ethnicity was grouped as ‘White’, ‘Black’, ‘Hispanic’ and ‘Other’. Educational attainment was defined as ‘no school‐leaving certificate’, ‘elementary’, ‘some high school’, ‘some college or technical school’ or ‘college graduate’. Smoking was categorised as ‘never’, ‘current’ and ‘former’. In a sensitivity analysis, since cigarette taxes might influence individual‐level alcohol consumption (Mostofsky et al. 2024), we additionally adjusted for cigarette taxes (federal and state tax per pack adjusted to 2012 dollars) from the Tax Burden on Tobacco (Orzechowski and Walker 2023). We excluded potential mediators, such as body mass index (BMI), from the primary regression equations to avoid bias in effect estimates (M in Figure 1). In secondary analyses, we examined effects on intermediate outcomes (BMI, dental visits) and a positive control outcome (coronary heart disease [CHD]). BMI was calculated as body weight (in kilograms) divided by squared body height (in metres). Dental visits were defined as ≥ 1 visit to a dentist during the past 12 months. Study participants were asked whether their doctor had told them they had CHD.

**TABLE 1:** Descriptive statistics of BRFSS data merged with state‐level alcohol taxes.

Number of participants	514,357
Edentulism	21,893 (4.3%)
Drinks per day	2.0 (1.1)
Drinks per day (categorical)	
> 0–2	302,147 (58.7%)
> 2 to < 5	200,653 (39.0%)
≥ 5	11,557 (2.2%)
Beer tax in 2012 US$ per gallon	0.6 (0.5)
Spirits tax in 2012 US$ per gallon	8.4 (4.5)
Wine tax in 2012 US$ per gallon	1.5 (1.1)
Sex	
Men	233,497 (45.4%)
Women	280,860 (54.6%)
Age, years	54.5 (14.2)
Race/ethnicity	
White	433,213 (84.2%)
Black	27,890 (5.4%)
Hispanic	28,905 (5.6%)
Other	24,349 (4.7%)
Education	
No school‐leaving certificate	329 (0.1%)
Elementary	6668 (1.3%)
Some high school	16,422 (3.2%)
High school graduate	121,815 (23.7%)
Some college or technical school	136,299 (26.5%)
College graduate	232,824 (45.3%)
Smoking status	
Never	249,943 (48.6%)
Current	90,964 (17.7%)
Former	173,450 (33.7%)
Cigarette pack tax in 2012 US$	4.4 (2.2)

*Note*: Entries are means (standard deviations) for continuous variable and numbers of observations (%) for categorical variables.

Abbreviation: BRFSS = Behavioural Risk Factor Surveillance Surveys.

### 2.2 Statistical Analysis

We used an IV probit model to estimate the effect of alcohol consumption on edentulism (Wooldridge 2010). The model first regresses the endogenous alcohol exposure on taxes and covariates using linear regression, then enters the predicted exposure values into a second‐stage probit model for edentulism. We reported IV probit coefficients with 95% confidence intervals (CI). Risk ratios (RRs) per standard deviation (SD) increment in the exposure variable were derived after marginal standardisation (Greenland 2004). This procedure involves estimating edentulism risk if the sample is set to high consumption (mean number of drinks per day plus one SD) and if the sample is set to the mean number of drinks per day. The average adjusted risk if exposed to high consumption divided by the average risk if exposed to mean consumption is the standardised RR. The 95% CI is obtained using 250 bootstrap replications. Prevalence ratios (PRs) from multivariable‐adjusted probit regression were provided as a comparison.

Valid IV probit estimates require (i) strong IV–exposure association, with first‐stage partial *F*‐statistic > 10 (relevance assumption); (ii) no uncontrolled common causes with the outcome (conditional exchangeability assumption); and (iii) IV affecting the outcome only through the exposure (exclusion restriction assumption) (Figure 1) (Baiocchi et al. 2014; Wooldridge 2010). Exchangeability and exclusion restriction are not directly testable, but they can be supported by sensitivity analyses. We conducted sensitivity analyses to study the robustness to conditional exchangeability and exclusion restriction violations and additional analyses to examine the effect of alcohol consumption on intermediate outcomes as well as positive and negative control outcomes. Since cigarette taxes could affect individual‐level alcohol consumption and violate exchangeability, we also adjusted for state‐level cigarette taxes. The IV model produces consistent estimates if the IV is uncorrelated with the first‐stage residual error (𝜀). We followed Nevo and Rosen (2012) and assumed (i) directional concordance of the potential correlation between IV and 𝜀, and the correlation between drinks per day and 𝜀, and (ii) that the correlation between IV and 𝜀 is less than the correlation between drinks per day and 𝜀. We used this set‐up to bound two‐stage least‐squares linear probability model coefficients and CIs.

IV models examining alcohol consumption's effects on intermediate outcomes (BMI, dental visits) and on CHD as a positive control were performed. Using a positive control outcome is useful to replicate established risk relations and to illustrate that these can be shown in the present dataset. We performed IV models for edentulism in participants aged ≤ 15 years as a negative control analysis (Davies et al. 2017; Pizer 2016). Specifically, the negative control population of younger individuals should be subject to similar potential unaccounted IV‐outcome confounding at the state level. Our reporting followed STROBE (Strengthening the Reporting of Observational Studies in Epidemiology).

## 3 Results

The validity of alcohol taxes as instruments was confirmed through first‐stage linear regression. The partial *F*‐statistics for instrument strength was 34.5 in the total sample, 10.1 in men and 36.6 in women (Table 2). The first‐stage linear regression coefficients for beer and spirits taxes with drinks per day were positive but inverse for wine tax. Each 1.1 drink increment yielded an RR for edentulism of 1.12 (95% CI: 1.09–1.16), with slightly stronger effects observed in women compared to men. The corresponding adjusted PR from a multivariable probit model was 1.28 (95% CI: 1.24–1.32) (Table 3). However, the Wald statistic of the IV probit model rejected the null hypothesis of no endogeneity, indicating that a multivariable probit regression produced biased estimates.

**TABLE 2:** IV probit model for the effect of drinks per day on edentulism.

	Full sample	Men	Women
Partial *F*‐statistic	34.5	10.1	36.6
Wald test of exogeneity	*Χ* ^2^ = 215.1, Prob > *Χ* ^2^ = < 0.001	*Χ* ^2^ = 116.5, Prob > *Χ* ^2^ = < 0.001	*Χ* ^2^ = 73.6, Prob > *Χ* ^2^ = < 0.001
First‐stage OLS coefficient (95% CI)			
Beer tax	−0.017 (−0.023 to −0.011)	−0.010 (−0.017 to −0.003)	−0.028 (−0.035 to −0.020)
Spirits tax	−0.003 (−0.004 to −0.002)	−0.003 (−0.005 to −0.002)	−0.003 (−0.004 to −0.002)
Wine tax	0.012 (0.009–0.015)	0.012 (0.007–0.017)	0.012 (0.008–0.015)
Second‐stage probit coefficient (95% CI)	0.887 (0.869–0.905)	0.766 (0.755–0.776)	1.009 (0.934–1.085)
Risk ratio (95% CI)	1.12 (1.09–1.16)	1.09 (1.05–1.14)	1.17 (1.13–1.22)

*Note*: Risk ratio per standard deviation increment in exposure derived from average predicted probabilities of an instrumental variable probit model. Models adjusted for age, sex, race/ethnicity, educational attainment and smoking status.

Abbreviations: CI = confidence interval; OLS = ordinary least‐squares.

**TABLE 3:** Multivariable‐adjusted prevalence ratios from probit regression for the association between drinks per day and edentulism.

	Prevalence ratio	95% confidence interval
Total sample	1.28	1.24–1.32
Men	1.23	1.20–1.28
Women	1.34	1.29–1.40

*Note*: Prevalence ratio per standard deviation increment in drinks per day derived from average predicted probabilities of a multivariable‐adjusted probit model. Models adjusted for age, sex, race/ethnicity, educational attainment and smoking status.

Analyses that additionally adjusted for cigarette taxes or used only beer and spirits taxes as IVs yielded similar estimates (Tables S1 and S2). In a sensitivity analysis that introduced a correlation of IVs with the first‐stage error, we found that lower and upper bounds for the coefficient and the 95% CI of a two‐stage least‐squares linear probability model excluded the null (Table S3). Alcohol consumption positively affected BMI, dental visits and CHD (Table S4). An IV probit model fit on individuals younger than 16 years showed no significant effect of alcohol consumption on edentulism (Table S4).

## 4 Discussion

The results of this study suggest that alcohol consumption affects edentulism. While this aligns with some existing research, the observational literature on alcohol consumption and tooth loss has produced mixed results. Some studies have reported positive associations, such as a cross‐sectional study among Kiriri Indians in Brazil that found a positive association between alcohol dependence and tooth loss (odds ratio (OR) = 2.49) (Pinto‐Filho et al. 2018). A cross‐sectional study in rural Wisconsin observed an association between consuming ≥ 4 drinks and tooth loss (OR = 1.44) (Klein et al. 2004). The VA Dental Longitudinal Study found a positive association between drinks per day and the number of teeth lost over a 10‐year period (Copeland et al. 2004) (rate ratio = 1.34). A study in Japan reported an increased risk of tooth loss over 4 years among individuals consuming > 20 g of ethanol per day (OR = 1.29) (Okamoto et al. 2006).

Conversely, other studies have reported inverse associations. The Baltimore Longitudinal Study of Aging found an inverse association between drinks per day and the number of teeth lost (rate ratio = 0.34) (Copeland et al. 2004). The Health and Retirement Study observed a lower odds of incident edentulism among drinkers compared to non‐drinkers (OR = 0.75) (Weintraub et al. 2019). A cross‐sectional analysis of the National Health and Nutrition Examination Survey (NHANES) found an inverse association between moderate alcohol consumption and edentulism (OR = 0.61) (Wu et al. 2011). These contradictory findings may be partially explained by the inclusion of former drinkers in some studies, leading to an underestimation of the true risk associated with alcohol consumption. This phenomenon has also been observed in research on alcohol and CVD. Early studies suggested a cardioprotective effect of moderate alcohol consumption (Klatsky 2010; Klatsky and Udaltsova 2013; Wannamethee and Shaper 1997). However, more recent evidence, including large‐scale cohort studies and Mendelian randomisation (MR) analyses, has challenged this hypothesis, demonstrating a linear relationship between alcohol consumption and increased CVD risk (Millwood et al. 2023; Nyberg et al. 2022; Wood et al. 2018).

Several mechanisms could mediate the effect of alcohol consumption on edentulism. In the present study, we examined BMI and dental visits as potential mediators. We found that alcohol consumption was positively associated with BMI. It is well established that alcohol increases body weight (Kwok et al. 2019; Traversy and Chaput 2015), which can lead to the development of type 2 diabetes (Ahmad et al. 2022) and may ultimately increase the risk of periodontitis (Botelho et al. 2022). In the BRFSS, alcohol consumption showed a positive effect on dental visits. Prior research suggested that the use of health services (including outpatient visits, hospitalisations and inpatient days) could either increase or decrease with higher amounts of alcohol consumed (Armstrong et al. 1998; Baumeister et al. 2006; Rice et al. 2000; Zarkin et al. 2004). Similar to our study, a previous analysis of NHANES found that the number of drinking days per week was positively correlated with dental services use (OR = 1.23) (Neff et al. 2010). Another cross‐sectional study conducted in Stockholm, Sweden, suggested a positive association between alcohol intake and dental visits (Jansson 2008).

Additional mediators that were not empirically tested in this study include periodontitis, dental trauma and oral hygiene. Observational studies have suggested that alcohol consumption increases the risk of periodontitis (Baumeister et al. 2025; Oliveira et al. 2022; Pulikkotil et al. 2020; Wang et al. 2016), which is a main cause of tooth loss (Aida 2021; Buchwald et al. 2013). Furthermore, an MR study using genetic variants as IVs for alcohol exposure also revealed an elevated periodontitis risk (Baumeister et al. 2021), corroborating the observational evidence. Heavy episodic drinking increases the risk of maxillofacial injuries causing tooth loss (Ahmed et al. 2022; Grocock 2018). Lastly, heavy alcohol consumption could disrupt regular toothbrushing behaviours, leading to poorer oral hygiene and increased gingival inflammation, periodontitis and tooth loss (Mizutani et al. 2015).

Several potential limitations should be considered. First, edentulism and alcohol consumption were self‐reported. If the measurement error of alcohol intake is correlated with edentulism, this could induce differential measurement error in a standard regression model. Fortunately, IV analysis addresses this by using an exogenous instrument correlated with the true value of the mismeasured exposure but uncorrelated with either the measurement error or the outcome's error term, ensuring consistent parameter estimation (Carroll 2006; Greene 2020). Second, the BRFSS did not allow the categorisation of important tooth loss indicators, such as functional dentition. Third, the IV models could not accommodate the examination of nonlinear dose–response relationships between alcohol consumption and edentulism, despite the possibility that such relationships exist. Fourth, the study used data representing the adult US population, and the generalisability of our findings to populations with different socio‐economic, cultural or ethnic characteristics may be limited. Furthermore, IV analysis yields a complier‐specific effect estimate that applies to alcohol consumers whose behaviour is influenced by the tax instrument (Clarke and Windmeijer 2012). This limits the generalisability of the effect estimate to the broader population of alcohol consumers. Fifth, BRFSS did not collect information on the reason for dental visits, which would have been helpful to further disentangle the mediating role of dental visits. Sixth, while we aimed to minimise exchangeability violations by conditioning on measured confounders, we cannot entirely rule out the possibility of IV exchangeability violations due to unadjusted confounders of the IV–outcome association or IV exclusion violations due to uncontrolled direct effects of the instrument on the outcome. Partial identification using bounds indicated that the association of unadjusted state‐level confounders with edentulism would need to be stronger than the association of drinks per day with edentulism to substantially alter our conclusions. Additionally, falsification testing in a negative control population of younger individuals revealed no evidence of IV assumption violations.

In conclusion, this IV study suggests that alcohol consumption increases the risk of edentulism. However, further evidence from well‐conducted observational and quasi‐experimental studies is necessary to establish a definitive causal role of alcohol in the development of edentulism.

## Author Contributions

Sebastian‐Edgar Baumeister contributed to conception, design, data analysis and interpretation, and drafted and critically revised the manuscript. Birte Holtfreter, Thomas Kocher and Gustavo G. Nascimento contributed to conception, design and data interpretation, and critically revised the manuscript. All authors gave final approval and agreed to be accountable for all aspects of the work.

## Ethics Statement

BRFSS was positively evaluated by CDC's and the State Health Department's institutional review boards.

## Conflicts of Interest

The authors declare no conflicts of interest.

## Supporting information


**Table S1.** Effect of alcohol consumption on edentulism with additional adjustment for state cigarette tax.
**Table S2.** Effect of drinks per day on edentulism using beer and spirits taxes as instruments.
**Table S3.** Bounds for two‐stage least‐squares linear probability model coefficient and 95% confidence interval for drinks per day and edentulism with correlation between instrument and first‐stage residuals.
**Table S4.** Effect of drinks per day on mediators, positive and negative control outcomes.

## Data Availability

The BRFSS data are accessible at https://www.cdc.gov/brfss/. Alcohol and cigarette tax data are available from https://alcoholpolicy.niaaa.nih.gov and https://data.cdc.gov/Policy/The‐Tax‐Burden‐on‐Tobacco‐1970‐2019/7nwe‐3aj9/about_data.
