# Supplementary material for: Capitalising on Tax Variation to Estimate the Effect of Alcohol Consumption on Edentulism
Source: J Clin Periodontol. 2025 May 6;52(8):1108–14. doi: 10.1111/jcpe.14176 (PMC12259400; doi:10.1111/jcpe.14176)
Supplement: Supplementary file 1 — Table S1. Effect of alcohol consumption on edentulism with additional adjustment for state cigarette tax. Table S2. Effect of drinks per day on edentulism using beer and spirits taxes as instruments. Table S3. Bounds for two‐stage least‐squares linear probability model coefficient and 95% confidence interval for drinks per day and edentulism with correlation between instrument and first‐stage residuals. Table S4. Effect of drinks per day on mediators, positive and negative control outcomes. [file JCPE-52-1108-s001.docx]

# **Capitalizing on tax variation to estimate the effect of alcohol consumption on edentulism**

Supplement

Supplementary Table 1 Effect of alcohol consumption on edentulism with additional adjustment for state cigarette tax

|  | **Full sample** | **Men** | **Women** |
| --- | --- | --- | --- |
| Partial F-statistic | 29.4 | 16.7 | 14.5 |
| Wald test of exogeneity | Χ²=101.3,  Prob>Χ²=<0.001 | Χ²=84.5,  Prob>Χ²=<0.001 | Χ²=27.2, Prob>Χ²=<0.001 |
| 1^st^ stage OLS coefficient (95% CI) |  |  |  |
| Beer tax | -0.043 (-0.056; -0.030) | -0.046 (-0.066, -0.025) | -0.040 (-0.055; -0.025) |
| Spirits tax | -0.002 (-0.003; -0.001) | -0.004 (-0.006, -0.002) | -0.001 (-0.002; 0003) |
| Wine tax | 0.016 (0.012; 0.019) | 0.023 (0.016; 0.030) | 0.009 (0.006; 0.0138) |
| 2^nd^  stage probit coefficient (95% CI) | 0.854 (0.816, 0.892) | 0.727 (0.697, 0.757) | 1.028 (0.915; 1.141) |
| Risk ratio (95% CI) | 1.12 (1.08, 1.16) | 1.09 (1.03; 1.16) | 1.17 (1.11; 1.24) |

OLS = ordinary least-squares. CI = confidence interval. Risk ratio per standard deviation increment in exposure derived from average predicted probabilities of an instrumental variable probit model. Models adjusted for age, sex, race/ethnicity, educational attainment, smoking status, state cigarette taxes

Supplementary Table 2 Effect of drinks per day on edentulism using beer and spirits taxes as instruments

|  | **Full sample** | **Men** | **Women** |
| --- | --- | --- | --- |
| Partial F-statistic | 37.5 | 7.3 | 44.6 |
| Wald test of exogeneity | Χ²=65.3,  Prob>Χ²=<0.001 | Χ²=39.6, Prob>Χ²=<0.001 | Χ²=24.2, Prob>Χ²=<0.001 |
| 1^st^ stage OLS coefficient (95% CI) |  |  |  |
| Beer tax | -0.011 (-0.017; -0.005) | -0.001 (-0.007; 0.005) | -0.021 (-0.029; -0.014) |
| Spirits tax | -0.002 (-0.003; -0.002) | -0.002 (-0.003; -0.001) | -0.002 (-0.003; -0.001) |
| 2^nd^  stage probit coefficient (95% CI) | 0.835 (0.775; 0.895) | 0.757 (0.729; 0.784) | 0.887 (0.706; 1.067) |
| Risk ratio (95% CI) | 1.12 (1.08; 1.16) | 1.09 (1.05; 1.14) | 1.17 (1.13; 1.23) |

OLS = ordinary least-squares. CI = confidence interval. Risk ratio per standard deviation increment in exposure derived from average predicted probabilities of an instrumental variable probit model. Models adjusted for age, sex, race/ethnicity, educational attainment, and smoking status.

Supplementary Table 3 Bounds for two-stage least-squares linear probability model coefficient and 95% confidence interval for drinks per day and edentulism with correlation between instrument and first-stage residuals

| **Coefficient [95% CI]** | **Coefficient bounds** | **95% CI bounds** |
| --- | --- | --- |
| 0.205 [0.153; 0.258] | 0.187; 0.224 | 0.099; 0.312 |

Models adjusted for age, sex, race/ethnicity, educational attainment, and smoking status.

Supplementary Table 4 Effect of drinks per day on mediators, positive and negative control outcomes

|  | **IV regression coefficient^1^ (95% CI)** |
| --- | --- |
| Body mass index (kg/m²) | 13.400 (10.353; 16.446) |
| Dental visits (yes/no) | 0.302 (0.223; 0.381) |
| Coronary artery disease (yes/no) | 0.596 (0.445; 0.749) |
| Edentulism in sample restricted to age <16 years (yes/no) | 0.344 (-0.484; 1.173) |

**^1^** Two-stage least-squares model: body mass index. IV probit model: dentist visits, coronary heart disease, edentulism.
